# Editorial

**Published:** 1869-11

**Authors:** 


### Editorial.

---

MEDICAL COLLEGES.—The two regular medical colleges in this city are again in active operation. The Rush Medical College opened its annual session on the last Wednesday in September. The introductory lecture was delivered by Prof. E. L. Holmes, who had recently been appointed to the newly created Chair of Ophthalmology.

This lecture was well written, and his advice to the students well received. With the exception of adding one or two professorships, on special departments, this school still adheres closely to the old arrangement of short terms, single classes, and heterogeneous teaching.

The Chicago Medical College, Medical Department of North-Western University, opened its annual session for 1866–70, on the first Monday in October. The general introductory lecture was given by Prof. W. H. Boyd, recently appointed to the Chair of Descriptive Anatomy. The address was highly appropriate, and was listened to with marked attention. It will be found at length in another part of this number of the EXAMINER. This school, although requiring three courses of lectures, of six months each, a consecutive order of studies, annual examinations, and some preliminary education, attracts an excellent class of students, and is practically demonstrating the feasibility of all the improvements in our system of medical education, recommended by the Convention of 1866, at Cincinnati, and so long demanded by the profession. No city in our country affords better advantages for the study of medicine in all its departments, didactically and clinically, than Chicago.

---

ITEMS FROM THE GAZETTE DE HOSPATEAUX, TRANSLATED BY F. H. DAVIS.—*Gazette of September 4th.* * * * We are happy to hear that the cholera has completely disappeared from the Province of Bengale.

* * The Typhus has been raging terribly for the last three months in Erzerum. One of our colleagues, Dr. Delort, Sanitary Physician, appointed by the French Government, has died a victim to his admirable devotion.

*Gazette, August 24th.* The *Bulletin* of the Prefecture gave a mortality of 75 cases, from affections of the digestive organs, in Paris, during the last week; diarrhœa 61; cholera and dysentery 5. The number of cases has apparently diminished, this week the *Bulletin* not indicating more than 4 deaths by cholera and 48 by diarrhœa.

* * The cholera, which has been prevailing with great violence in the western part of Africa, especially in Gambie, has completely depopulated that English colony.

* * The total number of victims at Sainte-Marie de Bathurst is estimated at 1323, from a population of 4000 to 4500, composed almost entirely of negroes and mulattoes; of the 25 whites who formed the little European colony, *one* only has been attacked by the disease, and he has fortunately been saved.

* * According to the last advices, the epidemic of cholera, which had been reported as prevailing in India, was much diminished, and apparently disappearing.

* * According to the *Gazette Medica de Bohia*, the number of consumptives in Bohia has been increasing for some time at a fearful rate. The journal attributes this increase to the imigration of foreign races, especialy the Germans, who have brought into Brazil the habits of *intemperance.*

When Brazil was colonized by the Portuguese alone, a race noted for their sobriety, tuberculosis was no more prevalent there than in Portugal. To-day the proportion is very different.

In mentioning the meeting of the Faculty of Medicine of Paris, held August 14th, the *Gazette* says:—"The standard of the students is not raising. This year, again, the Faculty of the three divisions of the Ecole pratique have not found a single student who merited a reward, or even a mention.

All the interest of the meeting was in the eulogy on Prof. Trousseau. M. Laséque has obtained the most legitimate success in committing to speech these remembrances. In listening, one could almost imagine that he saw again that grand and noble figure of a beloved master; and when he portrayed the last sufferings and death, all shared in the emotion which nearly suppressed his utterance with sobs.

"Oh no! it is not the students of the college who have changed. Restore to them their Trousseau, their Vulpean, and you will see that she has the same culture from them."

Prevention of the Spread of Cholera.—The French Government has sent Dr. Proust on an important mission to Persia. He is to explore the shores of the Caspean Sea, from Austrakan to Recht, with the view of ascertaining the local conditions which have caused the cholera always to follow that course, in extending from Persia into Europe; to study on the spot the measures taken by the Russian Government to prevent a fresh invasion of the disease, and to point out the means of more sure prevention.

The plan laid down for him is as follows:—After visiting St. Petersburg, and explaining the object of his mission, and being, perhaps, joined by a Russian physician, he will proceed to Austrakan, and visit the Russian quarantine establishments at that place. He will then explore the coast from Astrakan to Recht, and will thence proceed to Teheran. Having arrived in that city, he will impress on the Persian Government the necessity of carrying out the sanitary organization projected two years ago, especially the regular performance of the duties of the Council of Health, which was formed at that time, but which has remained almost a dead letter. One of the most important objects to be effected will be to put a stop, especially during the prevalence of cholera, to the carrying of dead bodies in a state of putrefaction with the pilgrims' caravans. This mission of M. Proust is highly important and well-timed; especially as it was reported in the middle of last month that cholera was prevailing at Teheran, and that there was some danger of its spreading along the shores of the Caspian Sea into Europe.—*British Medical Journal.*

---

### FOREIGN GLEANINGS.

M. Chairou has presented to the Academy of Medicine of Paris a memoir, having for its title "Clinical Studies upon the Nature and Coördination of Hysterical Phenomena."

The author is the Physician to the Asylum of Vésinet, and his conclusions, based upon his observations of the convalescents of that establishment, are expressed in the following propositions:

*1st.* Whenever, in a female, there is compression or inflammation of one or both ovaries, there is almost always a reflex sympathetic paralysis of the epiglottis and of all the organs constituting the pharynx.

*2d.* Whenever these two phenomena are found united in the same person, there is the beginning of a condition which the author calls the *hysterical cahexia.*

*3d.* The attack of hysteria is only the consequence of this reflex paralysis. The epiglottis fallen back over the superior orifice of the larynx cannot be raised by its muscular attachments and hence the attack of suffocation, the convulsive movements of the extremities and the general spasms constituting the hysterical convulsions.

*4th.* The asphyxia resulting from these repeated convulsions results necessarily in a perversion of vitality; and, as a consequence, those sensorial and anæsthetic disturbances almost always observed in hysterical patients.

*5th.* The treatment ought consequently to be addressed directly to the functional disorders of the ovaries; especially ought it to be local, to determine the resolution of the ovaries, the principal, if not the only, cause of the difficulty.

It is well-known that M. Auzias Turenne is an earnest student of the literature as well as the pathology and therapeutics of syphilis. In a note presented to the Academy of Medicine, at its meeting of September 7th, he expresses the following conclusions as to the origin of this disease in Europe. It is certain, according to M. Turenne, that syphilis, after having appeared in Spain, became epidemic first at Naples, in 1495, while that city was besieged by the French and defended by the Spaniards and Italians. The author discusses the different opinions as to its origin in Europe, and adopts that so well sustained by Astruc, and which regards it as an importation from America. In support of this opinion, he adduces three kinds of proof—historical, nosological, and philological. The historical proofs consist of the testimony of Oviédo, of Theret, and the circumstantial details furnished by Roderic Dias. The disease is followed chronologically and geographically from the West Indies through Spain to Italy, and thence to France, and its subsequent dispersion throughout the world.

The nosological proofs consist, 1st, in the well-known generalization and benignity of the disease in the West Indies, at the time of their discovery; 2d, in its march through Europe; at first insidious, then active and terrible, and, subsequently, gradually diminishing in its severity.

The philological proofs are, 1st, all the names of the disease and its symptoms are found in the Caribian dictionary and in the language of the Indians, to the exclusion of those of diseases which have been introduced into America by Europeans. 2d. the modern names of the disease are borrowed from the names of the people from whom each nation supposes it to have been derived. The Spanish called it the Indian Disease, the French, the Spanish Disease. The author, in accordance with his theories, already enunciated, concludes that the disease is of American origin, that its traditional treatment by mercury should be abandoned, and that syphilis itself is its proper remedy and preventive.

The discussion in the French Academy upon vaccination still rages, with all the virulence of genuine variola. We shall give the conclusions, if they are ever reached.

---

DISINFECTANT FOR PURPOSES OF DISSECTION.—All the bodies destined for dissection in the Ecole Pratique last winter were injected with a mixture of three litres of glycerine, and one quart of phenic acid; and the disinfection was as complete as could be desired.—*N. Y. Med. Record.*

---

A NEW METHOD OF PRESERVING PATHOLOGICAL SPECIMENS.—Dr. DeCamp, of Grand Rapids, Mich. (*Transactions of the Mich. State Med. Society*), gives us the following formula for preserving anatomical and pathological specimens:—

℞. Syrupus simp. (saturated strength); aqua, āā, oj.; alcohol, 95 per cent. f ℥iv.; acid, carbolic, ʒj. M.

The proportions of these have to be varied according to the transparency desired in the particular specimen being prepared. For most articles, the above formula is the nearest correct.

The specimen requires to have the blood removed by maceration (as it is soluble in this solution), or it will discolor the fluid.

---

TREATMENT OF RHEUMATIC FEVER.—Henry William Fuller, M.D., in a clinical lecture "On the Treatment of Rheumatic Fever" (*St. George's Hospital Reports*), gives us his treatment, and the subjoined statistics. From January 1st, 1845, to May 1st, 1848, 246 cases of acute rheumatism were admitted into St. George's Hospital, and they remained in the institution on the average 35 days; of these 246 patients, 119, or 1 in every 2.06, had some form of recent affection of the heart; and 1 in every 6.3 had pericarditis. During the six years ending December 31st, 1850, 17 cases, or about 1 out of every 27 cases of rheumatic fever admitted into the hospital terminated fatally. Since 1852, 417 cases of rheumatic fever have been treated by the alkaline treatment, and the disease did not prove fatal in a single instance. The alkalies should be given in such quantity that alkalinity of the urine will be induced within 24 to 36 hours. Dr. Fuller prefers the following prescription:—R. Potassæ acetatis, ʒss; sodæ carbonatis, ʒiss; aquæ, f ℥iij.; rendered effervescent by the addition of succus limonis, f ʒj. This to be taken at a draught, and repeated every fourth hour.—*N. Y. Med. Record.*

### Mortality for the Month of September, 1869:—

CAUSES OF DEATH,

| | |
|---|---|
| Accident, drowned | 5 |
| " by fall | 3 |
| " fracture of skull, | 1 |
| " run over by wagon | 3 |
| " thrown from buggy | 1 |
| " poisoning | 1 |
| " scalded | 1 |
| " railroad | 6 |
| " fall from window | 1 |
| " peritonitis, result of injury | 1 |
| Abscess, process, and dysentery | 1 |
| " lumbar | 1 |
| Anus, imperforate | 1 |
| Albuminuria and scarlet fever | 1 |
| Apoplexy | 1 |
| " and intemperance | 1 |
| Aneurism of femoral artery | 1 |
| Births, premature | 15 |
| " tedious | 1 |
| " stillborn | 37 |
| Bladder, paralysis of, and debility | 1 |
| Bowels, inflammation, | 12 |
| " obstruction, | 2 |
| Brain, congestion of | 8 |
| " compression of, | 1 |
| " and mesenterica glands, disease of, | 1 |
| " inflammation | 5 |
| " softening of | 5 |
| Bronchitis | 2 |
| " and asthma | 1 |
| " chronic | 1 |
| " capillary | 2 |
| Cancer | 1 |
| " of liver and spleen | 1 |
| " of thigh | 1 |
| Childbirth | 1 |
| Cholera infantum | 156 |
| " " and teething | 2 |
| " morbus | 4 |
| " " and gastritis | 1 |
| Chorea | 1 |
| Convulsions | 73 |
| " puerperal | 2 |
| Consumption | 30 |
| " and dysentery | 2 |
| Croup | 8 |
| " diphtheretic | 3 |
| " membranous | 2 |
| Cyanch trachealis | 1 |
| Debility | 2 |
| " general | 3 |
| Delirium tremens | 2 |
| Diabitis | 1 |
| Diarrhœa | 47 |
| " and convulsions | 6 |
| " and teething | 3 |
| " chronic | 19 |
| Diphtheria | 24 |
| Dropsy | 9 |
| Dysentery | 38 |
| " chronic | 4 |
| " typhoid | 4 |
| Emphysema pulmonum | 1 |
| Encephalitis | 2 |
| Entero-colitis | 5 |
| " chronic | 2 |
| Epilepsy and intemperance | 1 |
| Erysipelas | 1 |
| " and diarrhœa | 1 |
| Fever, congestive | 2 |
| " puerperal | 4 |
| " remittent | 1 |
| " scarlet | 28 |
| " " malignant, | 2 |
| " typhoid | 22 |
| " " neuralgia | 1 |
| Hæmoptysis | 1 |
| Heart, disease of | 3 |
| " hypertrophy of | 1 |
| " organic disease of | 1 |
| " paralysis of | 1 |
| " valvular disease | 2 |
| Hepatitis | 1 |
| Hydrothorax | 1 |
| Hydrocephalus | 17 |
| " acute | 5 |
| " chronic | 2 |
| Inanition | 13 |
| Intussusception | 1 |
| Ileo colitis | 1 |
| Ileus and cancer of stomach | I |
| Jaundice | 1 |
| Kidneys, Bright's dis. | 2 |
| Kidneys, chronic inflammation of | 1 |
| Laryngismus stridulus | 1 |
| Laryngitis | 3 |
| Liver, cirrhosis of | 1 |
| " cancer of | 1 |
| " tubercular abscess of | 1 |
| Lungs, congestion of | 2 |
| Manslaughter | 3 |
| Mouth, canker sore | 2 |
| Measles | 2 |
| " and cholera infantum | 1 |
| Meningitis | 5 |
| " cerebro-spinal, | 1 |
| " spinal | 2 |
| " tubercular | 2 |
| Old age | 7 |
| " and diarrhœa | 1 |
| Osophagus, stricture of | 1 |
| Œdema pulmonum | 2 |
| Paralysis | 2 |
| " general | 1 |
| Pericarditis | 2 |
| Peritonitis | 3 |
| Phrenitis | 2 |
| Pneumonia | 10 |
| " broncho | 1 |
| " typhoid | 1 |
| Purpuria hemorrhagia | 1 |
| Pyæmia | 2 |
| Rheumatism | 1 |
| Scrofula | 3 |
| Small-pox | 3 |
| Stomatitis, mercurial | 1 |
| Suicide | 4 |
| Syphilis, congenital | 1 |
| Tabes mesenterica, | 43 |
| Teething | 30 |
| " and convulsions, | 2 |
| Throat, ulcerated sore, | 1 |
| Trismus narcentium | 2 |
| Uterus, cancer of | 2 |
| Uræmia poisoning following puerperal convulsions | 1 |
| Vitality, deficient | 1 |
| Whooping-cough | 6 |
| " and cholera morbus | 1 |
| Unknown | 3 |
| Total | 814 |

COMPARISON.

Deaths in Sept., 1869,-- 814 | Deaths in Sept., 1868,--- 741 | Increase, --- 73

Deaths in Aug., 1869,------------- 1071 | Decrease, ------------------ 257

AGES.

| | | | | | |
|---|---|---|---|---|---|
| Under 1 | 320 | 20 to 30 | 36 | 70 to 80 | 11 |
| 1 to 3 | 231 | 30 to 40 | 36 | 80 to 90 | 6 |
| 3 to 5 | 48 | 40 to 50 | 27 | 90 to 100 | 2 |
| 5 to 10 | 36 | 50 to 60 | 30 | | |
| 10 to 20 | 18 | 60 to 70 | 13 | Total, | 814 |

| | | | | | |
|---|---|---|---|---|---|
| Males, | 426 | Females, | 388 | Total, | 814 |
| Single, | 702 | Married | 112 | Total, | 814 |
| White, | 801 | Colored, | 13 | Total, | 814 |

NATIVITY.

| | | | | | |
|---|---|---|---|---|---|
| Austria | 1 | England, | 8 | Newfoundland, | 1 |
| Atlantic Ocean | 1 | France, | 1 | Norway, | 8 |
| Bohemia, | 6 | Germany, | 69 | Sweden, | 32 |
| Canada, | 5 | Italy | 1 | Scotland, | 2 |
| Chicago, Native, | 139 | Holland | 2 | Switzerland | 2 |
| Chicago, Foreign, | 387 | Isle of Man, | 1 | Unknown, | 7 |
| U. S., other parts, | 88 | Ireland, | 49 | | |
| Denmark, | 2 | New Brunswick, | 2 | Total, | 814 |

MORTALITY BY WARDS FOR THE MONTH.

| Wards. | Mortality. | Pop. in 1868. | One death in |
|---|---|---|---|
| 1 | 3 | 9,094 | 3031⅓ |
| 2 | 20 | 13,074 | 653¾ |
| 3 | 29 | 15,076 | 520 |
| 4 | 35 | 17,796 | 508½ |
| 5 | 63 | 16,033 | 254½ |
| 6 | 57 | 13,083 | 229½ |
| 7 | 83 | 25,492 | 307⅛ |
| 8 | 58 | 15,813 | 272½ |
| 9 | 51 | 19,297 | 378⅓ |
| 10 | 12 | 12,925 | 1077 |
| 11 | 30 | 14,340 | 478 |
| 12 | 117 | 17,485 | 149½ |
| 13 | 35 | 11,164 | 319 |
| 14 | 58 | 14,839 | 255 5-6 |
| 15 | 57 | 21,078 | 369¾ |
| 16 | 18 | 15,465 | 859 |

| Mortality. | |
|---|---|
| Accidents, | 23 |
| Bridewell | 1 |
| County Hospital, | 24 |
| Home for Friendless, | 2 |
| Hospital Alexian Brothers, | 1 |
| Immigrants, | 23 |
| Mercy Hospital, | 3 |
| Marine Hospital, | 2 |
| Manslaughter, | 3 |
| St. Joseph Orphan Asylum, | 2 |
| Suicide, | 4 |
| Total, | 814 |

---

New Treatment for Tapeworm.—Dr. Surtel (Gaz. Méd. de Paris) has tried the following method:—He gives in one dose two-thirds of an ounce of ether, followed by an ounce of castor oil two hours afterwards. The worm is thus discharged entire, and no pain is caused by the treatment.

---

The Sulphites as Anthelmintics.—Dr. Roe, of Dublin, has satisfied himself of the efficacy of the sulphites, especially of soda, in cases of tape-worm. He gives children ten grains of bisulphite of soda three times daily, preceding the treatment by an alkali, and following it by a purgative.

Hypodermic Injection of Caffeine in Poisoning by Morphia.—Dr. Senneker communicates to the *St. Louis Med. Journal* a case of this kink, where the patient was in a dangerous condition. He injected a grain of pure caffeine hypodermically; and after having injected three grains, in ten minutes the patient quickly recovered.—*N. Y. Med. Record.*

Male Fern in Tænia.—Prof. Christison (Boston *Medical & Surgical Journal*) declares the ethereal extract of male fern better than kousso or any other remedy for tænia. He has never failed with it. He gives it in doses of from 18 to 24 grains in syrup or emulsion; and repeats it at the end of a month or six weeks, by way of making sure of his work. The worm never comes away alive.—*N. Y. Med. Record.*

Dr. James McNaughton has been elected President, and Dr. James H. Armesby, Professor of Surgery, by the faculty of the Albany Medical College, in place of Dr. March, deceased. —*Ibid.*

